# Extraordinary spin to orbital angular momentum conversion on guided zone plates

**DOI:** 10.1038/s41598-021-87456-9

**Published:** 2021-04-13

**Authors:** Pablo Acebal, Luis Carretero, Salvador Blaya

**Affiliations:** Department of Materials Science, Optics and Electronic Technology, Miguel Hernández University, Elche, Spain

**Keywords:** Nanoscience and technology, Optics and photonics

## Abstract

Focusing systems with high numerical aperture can be used to convert spin angular momentum into orbital angular momentum with efficiencies of 50%, while for low numerical apertures this conversion vanishes. In this paper, based on the properties of binary Fresnel zone plates, we propose a structure that is achieved by making an accurate selection of the width and the depth of the rings. This allows us to obtain a large increase in the spin to orbital angular momentum conversion of the resulting focusing fields, and it also has the special characteristic that the obtained conversion is higher for low numerical aperture structures, where standard focusing systems do not work. The ability of the system to perform this extraordinary conversion is demonstrated by FDTD methods and an analytical model developed using a combination of guided mode theory for the structure and Stratton–Chu diffraction theory.

## Introduction

Generation and control of the angular momentum (AM) of light has become an important research area in optics. Together with energy and linear momentum, angular momentum is one of the main properties of light and it has important applications in fields such as optical manipulation, telecommunications^1,2^ or photonics. In general, the angular momentum of light can be divided into two different contributions: orbital angular momentum (OAM), related to vortex charge, and spin angular momentum (SAM), related to light polarization. Each one can be measured independently, since, for example, in the interaction with particles each contribution produces different effects (SAM causes the rotation of the particle around its own axis, while OAM produces a rotation around the beam propagation axis). Thus, although total angular momentum is a magnitude that is conserved, there is an interest in studying the angular momentum transfer between both contributions, which produces complex polarization effects. In this sense, Spin to Orbital Angular Momentum conversion has received enormous attention^3^, and several methodologies have been proposed in order to control it. Basically there are two mechanisms that produce spin to orbital angular momentum conversion: the interaction of paraxial light with anisotropic media possessing certain azimuthal symmetries^4–7^, which is the basis of the Q-plates; and non-paraxial optical fields in locally isotropic media, which explains the conversion for the tight focusing of light by a high-numerical-aperture lens^8–11^ or Fresnel zone plate^12^, scattering by a small particle or apertures^13^, high numerical aperture imaging of small particles^14^ and cylindrical dielectric and metallic waveguides^15^.

**Figure 1:**
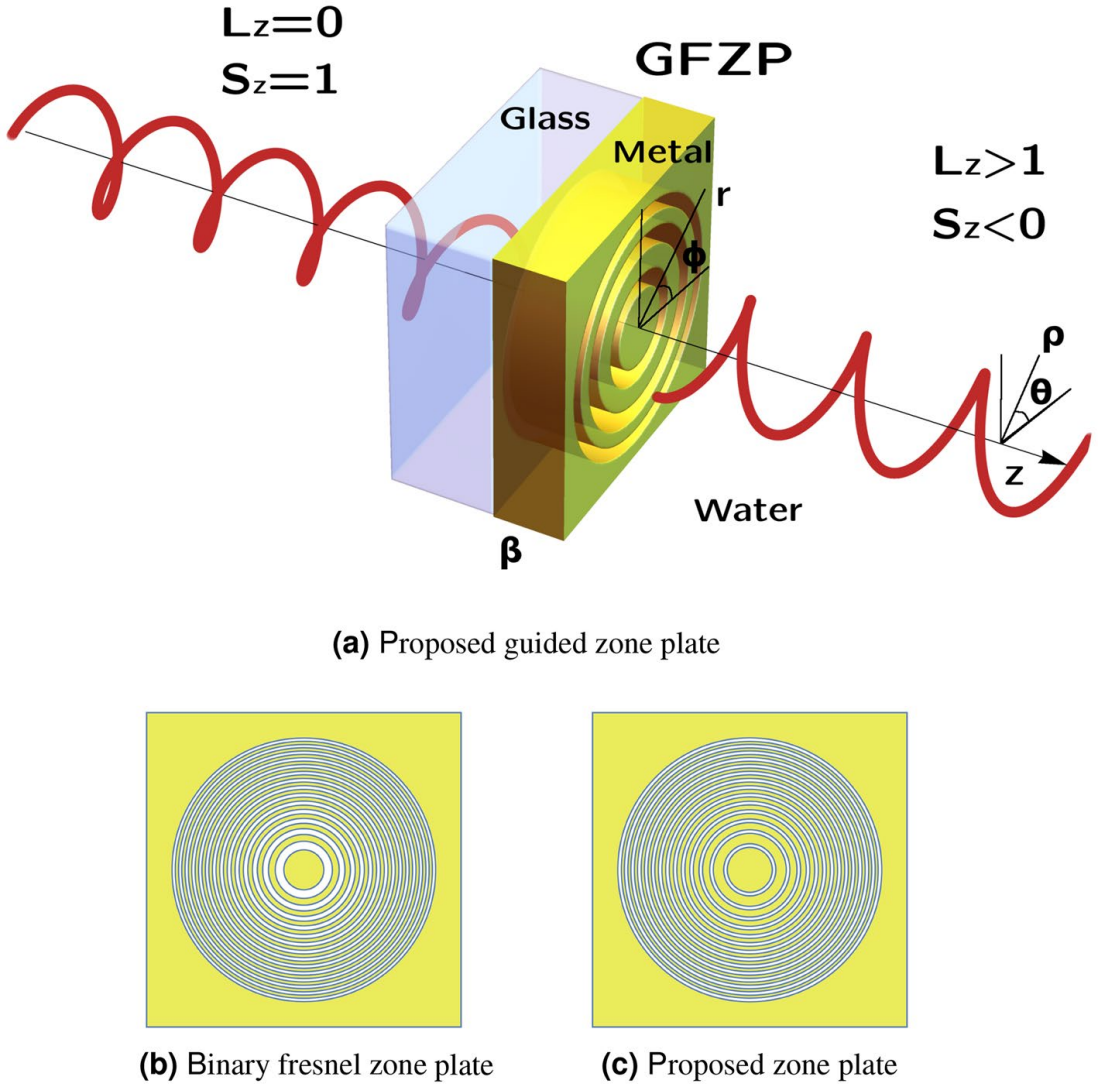
Schematic representation of the proposed system for spin to orbital angular momentum conversion. (**a**) SAM to OAM conversion in the proposed GFZP with the adequate selection of the width of the rings and the thickness $\beta$ of the structure. (r, $\phi$) denote cylindrical coordinates for the propagation in the structure. ($\rho$, $\theta$, z) cylindrical coordinates for the propagation in the homogeneous medium. (**b**, **c**) show the bidimensional cut of a standard binary zone plate and the proposed zone plate with equal widths.

Here, we propose the use of a modified binary Fresnel zone plate to achieve extraordinary spin to orbital angular momentum conversion. The proposed system, the so-called Guided Fresnel Zone Plate (GFZP), is shown in Fig. 1. We will demonstrate that if we carefully select the dimensions of the structure, it is possible to obtain an extraordinary spin to orbital angular momentum conversion, with efficiencies over 100% in the focus region. The proposed system is based on the characteristics displayed by the transmittance of small apertures^16^, which can be treated using waveguide theory. Therefore, in contrast to the standard binary zone plates (see Fig. 1b), which have different widths for each ring, in order to have complete control of amplitude and the propagation constants of the guided modes, the width of the different rings is the same, as shown in the schematic Fig. 1c. Moreover, the different propagation constants generate an anisotropy in cylindrical coordinates, which with the adequate selection of the thickness of the zone plate causes the extraordinary angular momentum conversion, with conversion efficiencies greater than Q-plates for low numerical aperture systems. It is important to note that, due to the employment of a binary structure, the energetic efficiency, defined as the transmitted power respect the incident one, can not be as high as that of Q-plate. However, our proposed system has other advantages respect to the Q-plates as: it is a passive element that can be adapted to different sizes (number of rings) and it will be easily employed on integrated systems due to the limited size; it is continuous in the azimuthal coordinate while the Q-plates are formed by discrete cells of liquid crystals with azimuthal pattern; it will be used in the near (few wavelengths) and the far field, since despite that the conversion efficiency is lower in the near field than for the far field, the values are much greater than that obtained from tightly focusing systems; and finally, may be the most important advantage, since our system is completely scalable to all the electromagnetic spectrum simply adapting the dimensions to the corresponding wavelength, from the RX to the microwaves, while the Q-plates are limited to the working spectra of the liquid crystals.

The paper is organized as follows. The theoretical background section includes three subsections that describe the electromagnetic properties investigated, the transmittance of the GFZP as a function of the propagating modes and finally the propagating fields in a homogeneous and dielectric medium calculated from approximations of the Stratton–Chu diffraction integrals. Results section includes a complete discussion of the results obtained for the proposed GFZP with a wide range of focal distances, including a comparison with FDTD calculations in order to validate the proposed procedure.

## Theoretical background

### Electromagnetic properties

The electromagnetic properties analyzed in this study are energy density (U), spin angular momentum (**S**), orbital angular momentum (**L**), canonical momentum (**P**), helicity ($\mathscr {H}$) and Poynting vector (${\mathbb {P}}$), which for a homogeneous and dielectric media can be written as^15^:

1 $$\begin{aligned} U= & {} \frac{1}{4}\left( \varepsilon {\mathbf {E}}^*\cdot {\mathbf {E}}+\mu {\mathbf {H}}^*\cdot {\mathbf {H}}\right) \end{aligned}$$

2 $$\begin{aligned} {\mathbf {S}}= & {} \frac{1}{4}\mathfrak {I}\left[ \varepsilon \left( {\mathbf {E}}^*\times {\mathbf {E}}\right) + \mu \left( {\mathbf {H}}^*\times {\mathbf {H}}\right) \right] \end{aligned}$$

3 $$\begin{aligned} {\mathbf {P}}= & {} \frac{1}{4}\mathfrak {I}\left[ \varepsilon {\mathbf {E}}^*\left( \nabla \otimes \mathbf {E}\right) +\mu {\mathbf {H}}^*\left( \nabla \otimes \mathbf {H}\right) \right] \end{aligned}$$

4 $$\begin{aligned} {\mathscr {H}}= & {} -\frac{1}{2}\sqrt{\varepsilon \mu } \mathfrak {I}\left[ {\mathbf {H}}^*\cdot {\mathbf {E}}\right] \end{aligned}$$

5 $$\begin{aligned} {\mathbb {P}}= & {} \frac{1}{2}\mathfrak {R}\left[ {\mathbf {E}}\times {\mathbf {H}}^*\right] \end{aligned}$$

Orbital angular momentum is ${\mathbf {L}}={\mathbf {r}}\times {\mathbf {P}}$, and total angular momentum is ${\mathbf {J}}={\mathbf {L}}+{\mathbf {S}}$. Here, **E** and **H** denote the electric and magnetic fields respectively, while $\varepsilon$ and $\mu$ are the dielectric constant and the magnetic permeability of the medium. Equation (1) and what follows the asterisk denote the complex conjugate, $\mathfrak {I}[]$ and $\mathfrak {R}[]$ being the imaginary and real part of the term between square brackets. The symbol $\otimes$ denote the dyadic product, so the operation ${\mathbf {W}}\nabla \otimes {\mathbf {V}}$ have the elements $({\mathbf {W}}\nabla \otimes {\mathbf {V}})_i=\sum _j\hbox {W}_j\partial _i\hbox {V}_j$ for i and j being {x, y, z}. In order to investigate the effect of the designed zone plate on the electromagnetic properties, we will calculate the above mentioned properties along with the normalized values *per photon*^15^, which are given by the local density ratios **S**/U, **L**/U or $\mathscr {H}/\hbox {U}$, and the corresponding integral ratios $\langle \mathbf {S}\rangle /\langle \hbox {U}\rangle$, etc.

### Zone plate transmittance: guided modes

In this section, we will derive the expressions of the electric and magnetic field at the output of the zone plate, which will be used later in the diffraction integrals. As mentioned above, the transmittance of small apertures can be explained by waveguide theory, so we will consider the transmittance zones of the GFZP as a set of coaxial waveguides, where the propagating modes basically depend on the width of each ring. For binary FZPs, the inner and outer radius of the transparent zones can be determined by equation $\hbox {r}_i=\sqrt{i \lambda f+ i^2 \lambda ^2}$, where $\lambda$ is the wavelength in the medium and f is the focal length of the zone plate. Therefore, the center of the different transparent zones is given by $\hbox {r}_{0i}=(\hbox {r}_{2i+2}+\hbox {r}_{2i+1})/2$, and the corresponding widths for standard binary FZPs $\hbox {w}_i=(\hbox {r}_{2i+2}-\hbox {r}_{2i+1})$, which implies different values for each ring (as can be seen in the schematic Fig. 1b). In the case of the proposed GFZP all the coaxial waveguides have been selected with the same width, so we can control the number and propagating characteristics of the modes allowed for all the waveguides. The characteristic solutions of the waveguides are TE and TM modes, which can be described with the following function and vectors in cylindrical coordinates (r, $\phi$, $\zeta$):

6 $$\begin{aligned} \vartheta= & {} Ae^{jk_z\zeta }e^{jm\phi }\left[ J_m\left( k_r r\right) +DY_m\left( k_r r\right) \right] \end{aligned}$$

7 $$\begin{aligned} \mathbf {v}_1= & {} \left\{ \frac{1}{r}\frac{\partial \vartheta }{\partial \phi },-\frac{\partial \vartheta }{\partial r},0\right\} \quad \mathbf {v}_2=\left\{ \frac{\partial \vartheta }{\partial r},\frac{1}{r}\frac{\partial \vartheta }{\partial \phi },-j\frac{k_r^2}{k_z}\vartheta \right\} \end{aligned}$$

where $\hbox {k}_z$ and $\hbox {k}_r$ are the components of the wavevector, z being the direction of propagation of the guide, while $\hbox {J}_m$ and $\hbox {Y}_m$ denote the corresponding Bessel functions of the first kind and second kind respectively. Therefore, the electric and magnetic fields for the TE modes are ${\mathbf {E}}_{TE}={\mathbf {v}}_1$ and ${\mathbf {H}}_{TE}=\hbox {k}_z/(\eta \hbox {k}){\mathbf {v}}_2$, where the allowed modes obey the following condition as a function of the inner ($\hbox {r}_1$) and outer ($\hbox {r}_2$) radius:

8 $$\begin{aligned} J^\prime _m\left( k_r r_1\right) Y^\prime _m\left( k_r r_2\right) -J^\prime _m\left( k_r r_2\right) Y^\prime _m\left( k_r r_1\right) =0 \end{aligned}$$

So it is possible to obtain $\hbox {k}_r$ of the propagating modes, and therefore $\hbox {k}_z$ through the relation $\hbox {k}^2=\hbox {k}_r^2+\hbox {k}_z^2$. The constant D can be obtained from the boundary condition $\hbox {E}_\phi =0$ at $\hbox {r}=\hbox {r}_1$ or $\hbox {r}=\hbox {r}_2$. In the same way, the electric and magnetic fields for the TM modes are ${\mathbf {E}}_{TM}={\mathbf {v}}_2$ and ${\mathbf {H}}_{TM}=-\hbox {k}/(\eta \hbox {k}_z)\mathbf{v} _1$, where $\hbox {k}_r$ is obtained from the following condition:

9 $$\begin{aligned} J_m\left( k_r r_1\right) Y_m\left( k_r r_2\right) -J_m\left( k_r r_2\right) Y_m\left( k_r r_1\right) =0 \end{aligned}$$

In this case the constant D can be obtained from the boundary condition $\hbox {E}_z=0$ at $\hbox {r}=\hbox {r}_1$ or $\hbox {r}=\hbox {r}_2$. Finally, the excited amplitudes $\hbox {A}_{mn}$ for each propagation mode under illumination conditions can be obtained from the continuity condition of the transverse components at the interface, depending on the incident electric and magnetic fields and are given by:

10 $$\begin{aligned} A_{mn}= & {} \frac{1}{2N_{mn}}\left( \displaystyle \iint _\Sigma \left( \left( \mathbf {h}_{mn}^*\times \mathbf {E}_i\right) \cdot {\hat{\zeta }}\right) r d\phi dr-\displaystyle \iint _\Sigma \left( \left( \mathbf {e}_{mn}^*\times \mathbf {H}_i\right) \cdot {\hat{\zeta }}\right) r d\phi dr\right) \end{aligned}$$

11 $$\begin{aligned} N_{mn}= & {} \displaystyle \iint _\Sigma \left( \left( \mathbf {h}_{mn}^*\times \mathbf {e}_{mn}\right) \cdot {\hat{\zeta }}\right) r d\phi dr \end{aligned}$$

where ${\mathbf {e}}_{mn}$ and ${\mathbf {h}}_{mn}$ are the transverse components of the different modes and $\Sigma$ indicates the dimensions of the waveguide.

**Figure 2:**
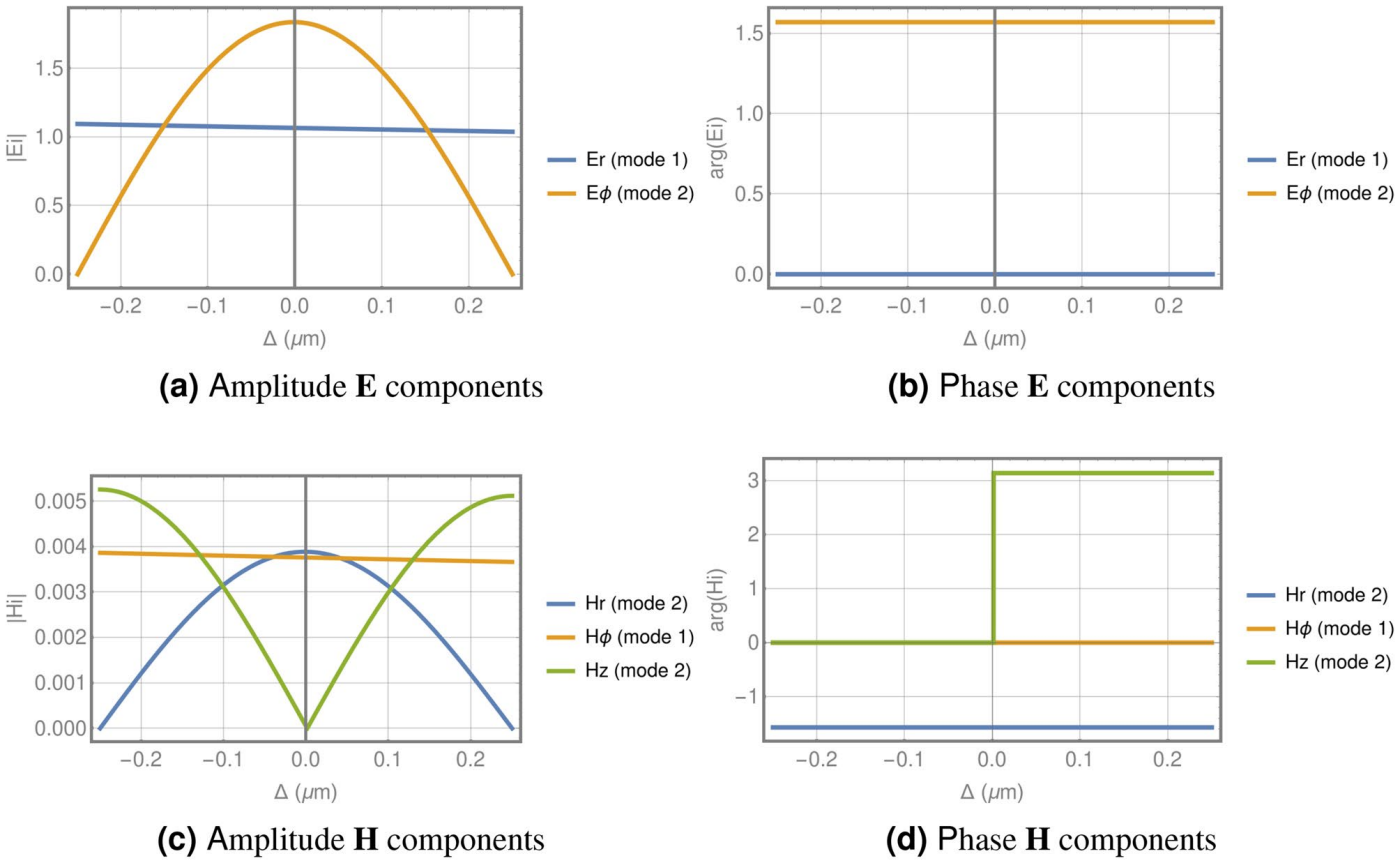
Amplitude and phase for the components of the electric and magnetic fields of the guided modes respect to the center of the guide

Let us consider a right circularly polarized plane wave $({\mathbf {E}}_i=\hbox {e}^{j(k\zeta +\phi )}\{1,j,0\}$ and ${\mathbf {H}}_i=\hbox {e}^{j(k\zeta +\phi )}/\eta \{-j,1,0\}$) with working wavelength in vacuum of $\lambda _0=1.064\,\upmu \hbox {m}$ illuminating a binary zone plate with 15 rings with a fixed width of $\hbox {w}_i=2\delta =0.5\,\upmu \hbox {m}$. Under these conditions, three modes can be propagated in each coaxial guide ($\hbox {TE}_{11}$, $\hbox {TE}_{12}$ and $\hbox {TM}_{11}$), but only the two TE modes are efficiently excited through the plane wave illumination. Figure 2 illustrates one waveguide (all of them have the same behaviour), showing the amplitude and phase of electric and magnetic field components as a function of the displacement with respect to the center of the guide at the beginning of the GFZP (all the components include a topological charge of 1 as the plane wave illumination). As can be seen, both modes are orthogonal, since $\hbox {TE}_{11}$ has radial component for the electric field with a constant amplitude of approximately 1, while $\hbox {TE}_{12}$ presents only azimuthal component for the electric field, with a spatial average amplitude around 1. Both components present a phase difference of $\pi /2$, so the electromagnetic field retains the polarization properties of the incident plane wave at the entry of the zone plate. However, the propagation constant is different for the two modes, that causes an *anisotropy* with azimuthal symmetry, so with the adequate selection of the thickness structure $\beta$, the polarization will change at the output of the waveguide, and consequently, the properties of the electromagnetic fields propagating in the surrounding dielectric media, including an extraordinary SAM to OAM conversion.

At this point, we can analyze the fields at the output of the waveguide in the circular basis, which can be expressed as function of the corresponding fields in the cylindrical basis as:

12 $$\begin{aligned} \mathsf {F}^{\pm }=\frac{e^{\pm j\phi }}{\sqrt{2}}\left( \mathsf {F}_{r}\pm j \mathsf {F}_{\phi }\right) \end{aligned}$$

where F denote the electric and magnetic components of the circular basis ($\hbox {F}^+$, $\hbox {F}^-$ and $\hbox {F}_z$). Since the width of the waveguides is less than half of the wavelength, for the diffraction integrals we are going to consider that the transmittance of the waveguide has a homogeneous distribution with the radial coordinate, so we use the spatial average as the amplitude for the components of the electric and magnetic fields. The values for average amplitude, initial phase and propagation constants of the different components are shown in Table 1. As can be seen, for the electric and magnetic field components, the amplitude and the initial phase difference are the same as the illumination conditions, but there is a phase difference of approximately $\pi$ between the propagation constants of the different modes, which will cause the mentioned anisotropy with cylindrical symmetry which depends on the thickness $\beta$ of the zone plate.

**Table 1:** Phase, propagation constant and spatial average of the amplitude for the components of electric and magnetic fields at the beginning of the waveguide.

	Amplitude	Phase ($\zeta =0$)	$\hbox {k}_z$ ($\upmu \hbox {m}^{-1}$)
$\hbox {E}_{r}$	1.067 V/m	0	7.85325
$\hbox {E}_{\phi }$	1.17 V/m	$\pi$/2	4.71026
$\hbox {E}_{z}$	0	0	–
$\hbox {H}_{r}$	0.00376 A/m	$-\pi$/2	4.71026
$\hbox {H}_{\phi }$	0.00247 A/m	0	7.85325
$\hbox {H}_{z}$	0.0033 A/m	0	4.71026

Therefore, the electric and magnetic field components in the circular basis at the output each ring of the GFZP with thickness $\beta$ can be expressed as:

13 $$\begin{aligned} \mathsf {E}^{\pm }_\beta= & {} \frac{e^{(1\pm 1) j\phi }}{\sqrt{2}}e^{jk_{z1}\beta }\left( |\mathsf {E}_{r}|\mp | \mathsf {E}_{\phi }|e^{j\Delta k_z \beta }\right) \end{aligned}$$

14 $$\begin{aligned} \mathsf {H}^{\pm }_\beta= & {} -j\frac{e^{(1\pm 1) j\phi }}{\sqrt{2}}e^{jk_{z1}\beta }\left( |\mathsf {H}_{r}|\mp | \mathsf {H}_{\phi }|e^{j\Delta k_z \beta }\right) \end{aligned}$$

15 $$\begin{aligned} \mathsf {H}_{z\beta }= & {} |H_{z}|e^{jk_{z2}\beta } \end{aligned}$$

where $\Delta \hbox {k}=\hbox {k}_{z2}-\hbox {k}_{z1}$. Finally, the electromagnetic fields at the output of the structure can be calculated taking into account the transmittance functions of the zone plate structure given by:

16 $$\begin{aligned} T_1^{GFZP}= & {} \sum _i \Theta \left[ r-\left( r_{0i}-\delta \right) \right] \Theta \left[ (r_{0i}+\delta )-r\right] \end{aligned}$$

17 $$\begin{aligned} T_2^{GFZP}= & {} \sum _i \Theta \left[ r-\left( r_{0i}-\delta \right) \right] \Theta \left[ \left( r_{0i}+\delta \right) -r\right] \left( 2\Theta \left[ r-r_{0i}\right] -1\right) \end{aligned}$$

where $\Theta$ denote the Heaviside function, which is used to limit the transparent zones and also in the case of the $\hbox {T}_2^{GFZP}$ take into account that $\hbox {H}_z$ component determines parity with respect to the center of the waveguides. Therefore, the electric and magnetic fields at the output of the GFZP, that we use in the next section as boundary conditions for the propagation integrals, are given by:

18 $$\begin{aligned} \mathsf {F}^{\pm }_0=\mathsf {F}^{\pm }_\beta T_1^{GFZP}\quad \mathsf {H}_{z0}=\mathsf {H}_{z\beta } T_2^{GFZP} \end{aligned}$$

where **F** can be **E** or **H**.

### Stratton–Chu diffraction theory

According to Strantton and Chu^17^, the electromagnetic field diffracted by an aperture can be obtained from the following diffraction formulae, which depends on the electric and magnetic field at the output plane of the object (${\mathbf {E}}_0$ and ${\mathbf {H}}_0$ respectively)

19 $$\begin{aligned} \mathbf {E}= & {} -\frac{1}{4\pi }\left[ \frac{1}{j\omega \varepsilon }\oint \nabla \psi \mathbf {H}_0\cdot d\mathbf {l} + \int _\Sigma \left( j\omega \mu \left( \mathbf {n}\times \mathbf {H}_0\right) \psi + \left( \mathbf {n}\times \mathbf {E}_0\right) \times \nabla \psi +\left( \mathbf {n}\cdot \mathbf {E}_0\right) \nabla \psi \right) d\Sigma \right] \end{aligned}$$

20 $$\begin{aligned} \mathbf {H}= & {} \frac{1}{4\pi }\left[ -\frac{1}{j\omega \mu }\oint \nabla \psi \mathbf {E}_0\cdot d\mathbf {l} + \int _\Sigma \left( j\omega \varepsilon \left( \mathbf {n}\times \mathbf {E}_0\right) \psi - \left( \mathbf {n}\times \mathbf {H}_0\right) \times \nabla \psi -\left( \mathbf {n}\cdot \mathbf {H}_0\right) \nabla \psi \right) d\Sigma \right] \end{aligned}$$

where $\hbox {R}=\sqrt{(x-x_0)^2+(y-y_0)^2+(z-z_0)^2}$ denotes the distance between the point of observation (x,y,z) and the points in the exit plane ($\hbox {x}_0$, $\hbox {y}_0$, $\hbox {z}_0$), while $\psi$ is given by

21 $$\begin{aligned} \psi =\frac{e^{jkR}}{R} \end{aligned}$$

where the observation plane is not too close to the exit plane, the gradient of $\psi$ function can be approximated to:

22 $$\begin{aligned} \nabla \psi \approx -jk\frac{e^{jkR}}{R^2} \left\{ \left( x-x_0\right) ,\left( y-y_0\right) ,\left( z-z_0\right) \right\} \end{aligned}$$

Due to the circular symmetry of the problem, it is more convenient to work with diffraction integrals in the circular basis, so we define the orthogonal components for electric field as $\hbox {E}^{-}=1/\sqrt{2}(\hbox {E}_x-\hbox {jE}_y)$, $\hbox {E}^{+}=1/\sqrt{2}(\hbox {E}_x+\hbox {jE}_y)$, and in the same way for the magnetic field $\hbox {H}^-$ and $\hbox {H}^+$. It is also appropriate to use polar coordinates rather than Cartesian for the integrals, so we use the following relations $\hbox {x}_0=\hbox {rCos}(\phi )$, $\hbox {y}_0=\hbox {rSin}(\phi )$, $\hbox {z}_0=0$, $\hbox {x}=\rho \hbox {Cos}(\theta )$ and $\hbox {y}=\rho \hbox {Cos}(\theta )$. Therefore, the diffraction integrals, assuming that the contribution of the line integral vanishes, can be expressed as:

23 $$\begin{aligned} \mathsf {E}^\pm= & {} \displaystyle \iint _\Sigma \frac{e^{jkR}}{4\pi R^2}k\left[ -j z \mathsf {E}_0^\pm \mp \eta R \mathsf {H}_0^{\pm }+j\frac{\mathsf {E}_{z0}}{\sqrt{2}}\left( e^{\pm j\phi }r-e^{\pm j\theta }\rho \right) \right] r dr d\phi \end{aligned}$$

24 $$\begin{aligned} \mathsf {E}_z= & {} \displaystyle \iint _\Sigma -jk\frac{e^{jkR}}{4\pi R^2}\left[ \mathsf {E}_{z0} z + \frac{\mathsf {E}^+_0}{\sqrt{2}} \left( e^{-j\phi }r - e^{-j\theta } \rho \right) + \frac{\mathsf {E}^-_0}{\sqrt{2}} \left( e^{j\phi }r - e^{j\theta } \rho \right) \right] r dr d\phi \end{aligned}$$

25 $$\begin{aligned} \mathsf {H}^\pm= & {} \displaystyle \iint _\Sigma \frac{e^{jkR}}{4\pi R^2}k\left[ -j z \mathsf {H}_0^\pm \pm \frac{R}{\eta } \mathsf {E}_0^{\pm }+j\frac{\mathsf {H}_{z0}}{\sqrt{2}}\left( e^{\pm j\phi }r-e^{\pm j\theta }\rho \right) \right] r dr d\phi \end{aligned}$$

26 $$\begin{aligned} \mathsf {H}_z= & {} \displaystyle \iint _\Sigma -jk\frac{e^{jkR}}{4\pi R^2}\left[ \mathsf {H}_{z0} z + \frac{\mathsf {H}^+_0}{\sqrt{2}} \left( e^{-j\phi }r - e^{-j\theta } \rho \right) + \frac{\mathsf {H}^-_0}{\sqrt{2}} \left( e^{j\phi }r - e^{j\theta } \rho \right) \right] r dr d\phi \end{aligned}$$

Now, we can use the guided modes calculated in the previous section as the initial fields for the diffraction problem. Analytic solutions of diffraction integral in each transparent zone can be obtained after some approximations involving the R function. On the one hand, for the coefficient R, which is not in the complex exponential function, we use $\hbox {R}\approx \hbox {R}_{0i}=\sqrt{r_{0i}^2+z^2}$ for each transparent zone, where $\hbox {r}_{0i}$ denotes the center of the rings that compose the zone plate. On the other hand, the R in the exponential function is approximated with series expansion at $\rho =0$ truncated in the first term:

27 $$\begin{aligned} R=\sqrt{\rho ^2+r^2-2 \rho r \cos (\theta -\phi )+z^2}\approx \sqrt{r^2+z^2}-\frac{r \rho }{\sqrt{r^2+z^2}}Cos(\theta -\phi ) \end{aligned}$$

With these approximations, it is possible to integrate over the $\phi$ variable with the expansion in Bessel functions of the exponential of Cos($\theta -\phi$). In the case of the r variable, we use a similar approximation to that of R, assuming that $\hbox {r}\approx \hbox {r}_{0i}$ except for the case of the exponential that is assumed to be:

28 $$\begin{aligned} e^{jk\sqrt{r^2+z^2}}\approx e^{jkR_{0i}}e^{jk\frac{r_{0i}}{R_{0i}}\left( r-r_{0i}\right) } \end{aligned}$$

Therefore, analytical integration over the r variable in the different transparent zones can be done. The resulting electric and magnetic fields are given by:

29 $$\begin{aligned} \mathsf {E}^-= & {} -jz \Gamma _{0,0}^{(1)} \mathsf {E}^-_\beta +\eta \Gamma _{0,0}^{(0)} \mathsf {H}^-_\beta \end{aligned}$$

30 $$\begin{aligned} \mathsf {E}^+= & {} e^{2j\theta }\left( jz \Gamma _{2,0}^{(1)} \mathsf {E}^+_\beta +\eta \Gamma _{2,0}^{(0)} \mathsf {H}^+_\beta \right) \end{aligned}$$

31 $$\begin{aligned} \mathsf {E}_z= & {} \frac{1}{\sqrt{2}}e^{j\theta }\left( - \left( \mathsf {E}^-_\beta +\mathsf {E}^+_\beta \right) \Gamma _{1,1}^{(1)} +j \rho \left( \Gamma _{0,0}^{(1)} \mathsf {E}^-_\beta - \Gamma _{2,0}^{(1)} \mathsf {E}^+_\beta \right) \right) \end{aligned}$$

32 $$\begin{aligned} \mathsf {H}^-= & {} -\frac{1}{\eta }\Gamma _{0,0}^{(0)} \mathsf {E}^-_\beta -jz\Gamma _{0,0}^{(1)} \mathsf {H}^-_\beta +\frac{\mathsf {H}_{z\beta }}{\sqrt{2}}\left( j\Lambda _{0,1}^{(1)}-\rho \Lambda _{1,0}^{(1)}\right) \end{aligned}$$

33 $$\begin{aligned} \mathsf {H}^+= & {} e^{2j\theta }\left( -\frac{1}{\eta }\Gamma _{2,0}^{(0)} \mathsf {E}^+_\beta + jz\Gamma _{2,0}^{(1)} \mathsf {H}^+_\beta -\frac{\mathsf {H}_{z\beta }}{\sqrt{2}}\left( j\Lambda _{2,1}^{(1)}+\rho \Lambda _{1,0}^{(1)}\right) \right) \end{aligned}$$

34 $$\begin{aligned} \mathsf {H}_z= & {} \frac{1}{\sqrt{2}}e^{j\theta }\left( - \left( \mathsf {H}^-_\beta +\mathsf {H}^+_\beta \right) \Gamma _{1,1}^{(1)} + j \rho \left( \Gamma _{0,0}^{(1)} \mathsf {H}^-_\beta - \Gamma _{2,0}^{(1)} \mathsf {H}^+_\beta \right) -\sqrt{2} z \Lambda _{1,0}^{(1)} \mathsf {H}_{z\beta } \right) \end{aligned}$$

where the $\Gamma$ and $\Lambda$ functions are:

35 $$\begin{aligned} \Gamma _{\alpha 1,\alpha 2}^{(\alpha 3)}= & {} \displaystyle \sum _i e^{jk R_{0i}} \mathsf {J}_{\alpha 1}\left[ k\frac{r_{0i}}{R_{0i}} \rho \right] r_{0i}^{\alpha 2} R_{0i}^{\alpha 3} Sin\left[ k\frac{r_{0i}}{R_{0i}}\delta \right] \end{aligned}$$

36 $$\begin{aligned} \Lambda _{\alpha 1,\alpha 2}^{(\alpha 3)}= & {} -2j\displaystyle \sum _i e^{jk R_{0i}} \mathsf {J}_{\alpha 1}\left[ k\frac{r_{0i}}{R_{0i}} \rho \right] r_{0i}^{\alpha 2} R_{0i}^{\alpha 3} \left( 1-Cos\left[ k\frac{r_{0i}}{R_{0i}}\delta \right] \right) \end{aligned}$$

where the resulting electromagnetic fields propagating in the free space depend on two design parameters given by the GFZP, the focal point f, which determines $\hbox {r}_{0i}$, and depth d.

## Results and discussion

In this section, we are going to show the different electromagnetic properties for the fields generated with the GFZP as a function of design parameters, such as the focal length and structure thickness, paying special attention to the extraordinary spin-to-orbital angular momentum conversion. The effect of the guiding characteristics of the structure is studied for two different values of the structure depth: one with $\beta =0.1\,\upmu \hbox {m}$, which is about ten times lower than the wavelength, the so-called short guided Fresnel zone plate (SGFZP); and the other with $\beta =1\,\upmu \hbox {m}$, which we call the long guided Fresnel zone plate (LGFZP). A wide range of focal lengths is also analyzed to study the ability of the structure to perform the SAM to OAM conversion in the near or far field. In order to validate the approximations made in the analytical model, we also compare the results obtained with numerical simulations using the FDTD method (Meep program^18^) for various cases with values of focal distance from 3$\lambda$ to 9$\lambda$ (which implies a numerical aperture AN range from 1.22 for $\hbox {F}=3\lambda$ to 0.82 for $\hbox {F}=9\lambda$), where the metal selected to form the zone plate is gold, BK7 glass for the substrate and water for the propagating medium.

### Electromagnetic energy density and energy flux

**Figure 3:**
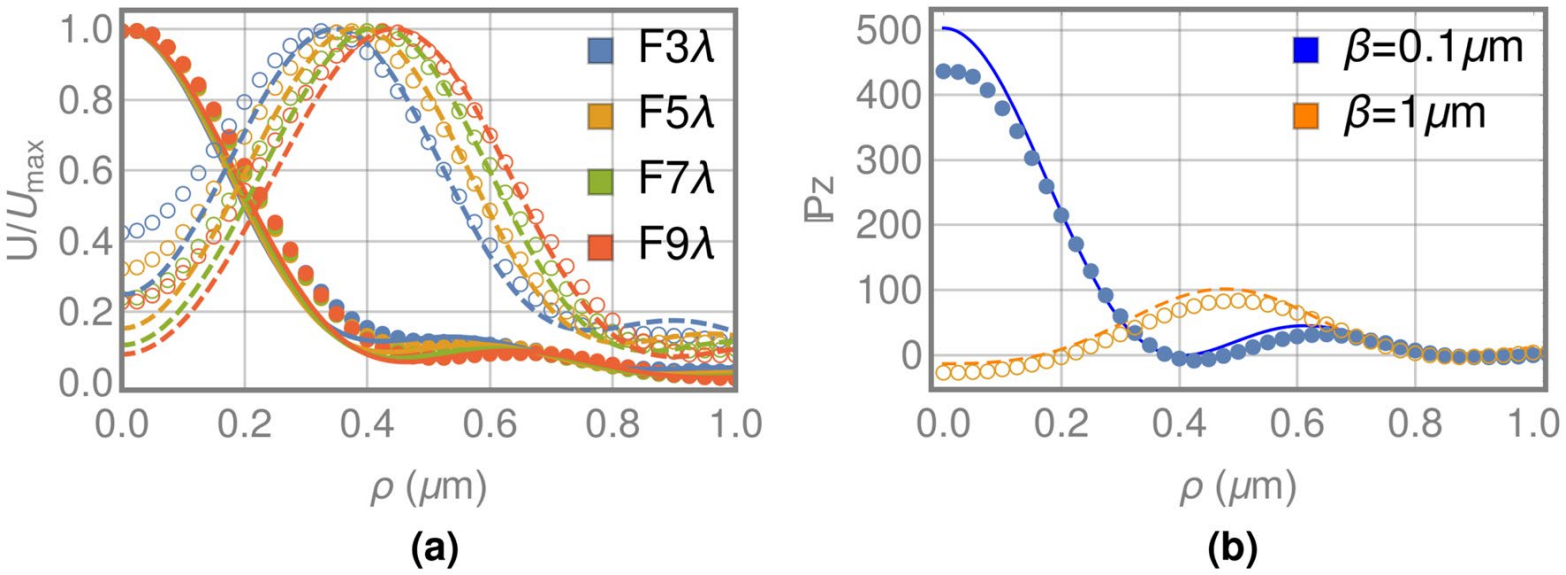
(**a**) Normalized electromagnetic energy in the focal plane for SGFZPs (continuous lines and filled circles) and LGFZPs (dashed lines and unfilled circles) with different focal lengths estimated through the proposed model (lines) and FDTD (circles). (**b**) Axial component of the Poynting vector in the focal plane for a SGFZP (continuous line and filled circles) and a LGFZP (dashed line and unfilled circles) with focal length of 7$\lambda$ estimated through the proposed model (lines) and FDTD (circles).

The results obtained for the normalized electromagnetic energy density U in the focal plane are shown in Fig. 3a for SGFZPs and LGFZPs with four different focal distances, calculated with the analytical model and the numerical FDTD method. For the SGFZP ($\beta =0.1\,\upmu \hbox {m}$) we can observe a behaviour that is similar to the characteristic airy disk of focusing systems, with a full width at half maximum (FWHM) of about $0.46\,\upmu \hbox {m}$, similar to the experimental values of reference^19^ for chromium FZPs with thickness between 0.06 and 0.09 $\upmu$m. Also it is important to note the good correlation between the analytical model presented and the results of the FDTD. An interesting result is obtained for the LGFZPs ($\beta =1\,\upmu \hbox {m}$), where the electromagnetic energy presents an annular distribution, whose maximum is shifted to a higher radius as the focal distance increases. As in the previous case, a good correlation has been observed between our model and the numerical FDTD results.

The strong influence of the depth of the structure on the focused electromagnetic energy as a consequence of the guiding characteristic is also observed for the other electromagnetic properties, resulting in very interesting behaviours for practically all of them. This is the case for the axial component of the Poynting vector, whose behaviour in the focal plane is shown in Figure 3b for one SGFZP and one LGFZP with a focal length of 7$\lambda$. The axial component of the Poynting vector ${\mathbb {P}}_z$ for the SGFZP shows the typical distribution of focusing systems, since the energy flows in the propagation direction with the same spatial distribution as electromagnetic energy. However, for the LGFZP, we observe that the energy flows in the direction of propagation in the region of the maximum values of U (annular distribution), but there is a small region near the axis where the ${\mathbb {P}}_z$ takes negative values, i.e., the energy flows opposite to the propagation direction, in the same way as described in reference^20^ for the focusing of circularly polarized optical vortex beams with topological charge 2 using Richards and Wolf formalism. The ratio between the negative flux in the axis and the maximum positive flux of the annular distribution increases as the focal length decreases, and for focal lengths greater than 23$\lambda$ the effect disappears, so the energy flux in the propagation direction become positive for all the positions.

### Helicity and angular momentum

**Figure 4:**
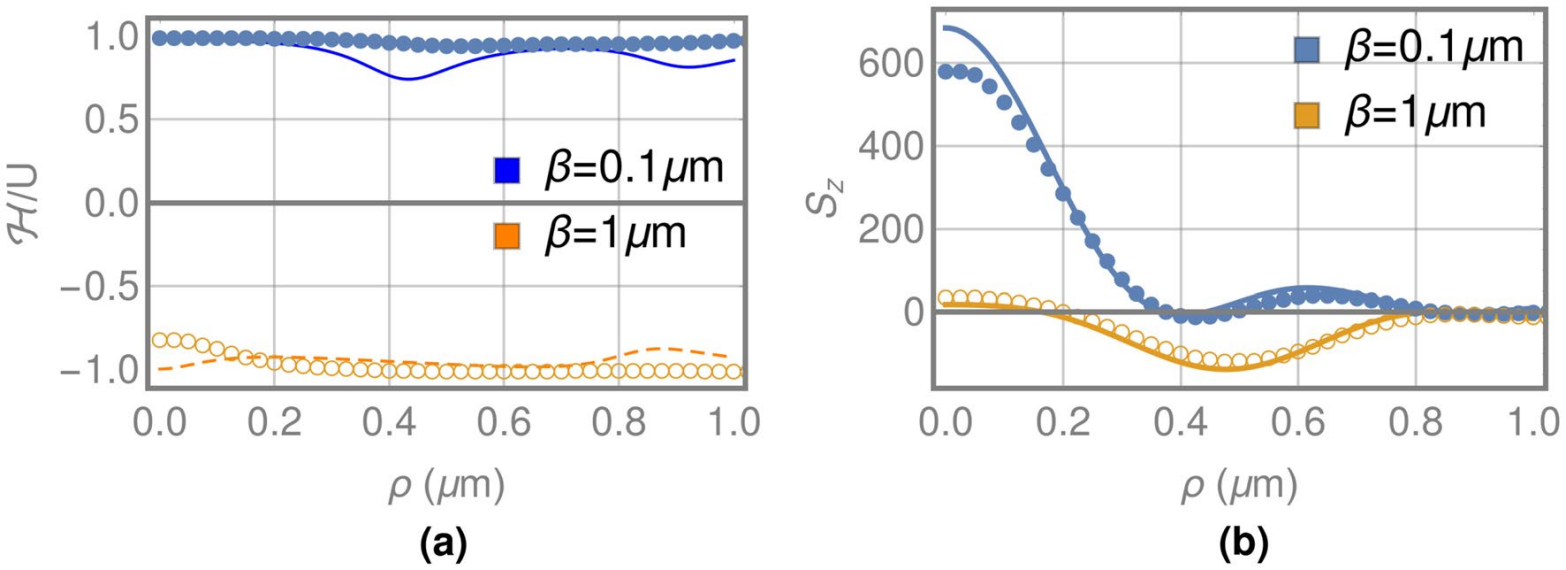
(**a**) Local density ratio of helicity in the focal plane for a SGFZP (continuous line and filled circles) and a LGFZP (dashed line and unfilled circles) with focal length of 7$\lambda$ estimated through the proposed model (lines) and FDTD (circles). (**b**) Axial component of the SAM in the focal plane for a SGFZP (continuous line and filled circles) and a LGFZP (dashed line and unfilled circles) with focal length of 7$\lambda$ estimated through the proposed model (lines) and FDTD (circles).

Finally, we are going to analyze the effect on two important electromagnetic properties: electromagnetic helicity, which is a measure of the chirality of the field, and in our case, it also has a strong influence on the thickness of the GFZP; and the axial component of the angular momentum, which can be divided into SAM and OAM contributions, where the conversion between them is strongly modified by the depth of structure. These properties are well defined for the incident beam, since the right circularly polarized plane wave presents an average value per photon of helicity ${\mathscr {H}}/U=1$. In the same way, the local density ratios for the axial component of the total and the spin angular momentum of the incident field are ${\mathbf {S}}_z/\hbox {U}={\mathbf {J}}_z/\hbox {U}=1$, since ${\mathbf {L}}_z/\hbox {U}=0$. Figure 4a shows the local density ratio of helicity in the focal plane for the SGFZP and LGFZP with a focal length of 7$\lambda$. On the one hand, for the SGFZP the value of ${\mathscr {H}}/\hbox {U}$ is about 1 for all the range of positions, so in this case the structure preserves the helicity of the incident fields. On the other hand, a complete flip of the ${\mathscr {H}}/\hbox {U}$ value is obtained for the fields generated with the LGFZP, where the value is around -1 for all the positions, so the structure generates a complete inversion of the chirality of the fields.

Figure 4b shows the behaviour of the axial component of the SAM ${\mathbf {S}}_z$ for both structures, SGFZP and LGFZP. As for helicity, the axial component of the SAM retains the same sign as the incident field for the SGFZP structure, which corresponds to the behavior of Fresnel zone plates or standard focusing systems. However, the LGFZP structure generates a change in the sign of ${\mathbf {S}}_z$ in the annular region where the energy is mainly focalized (see Fig. 3), being only slightly positive in the region near the axis. Therefore, as a consequence of the conservation of the angular momentum ${\mathbf {J}}_z$, this change in the sign of ${\mathbf {S}}_z$ will cause a large increase in the SAM to OAM conversion. We also want to point out that for both helicity and ${\mathbf {S}}_z$ there is a good correlation between the proposed model and the results of the FDTD, as in the case of the other electromagnetic properties.

**Figure 5:**
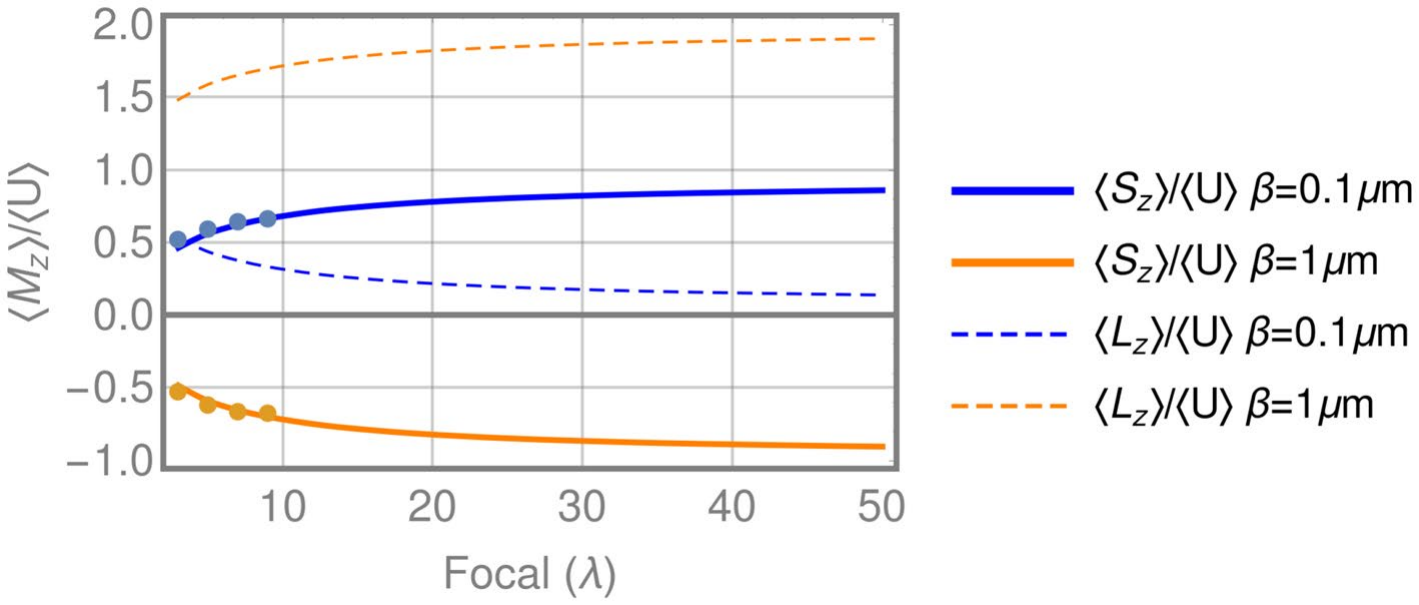
Evolution of SAM and OAM in the focal plane generated by SGFZP and LGFZP as a function of the focal length of the zone plate (points indicate the results of FDTD).

The SAM to OAM conversion is finally analyzed through the corresponding integral ratios of the axial component of the spin and orbital angular momentum in the focal plane, which can be considered the values per photon. In this way, Fig. 5 shows the integral ratios $\langle {\mathbf {S}}_z\rangle /\langle U\rangle$ and $\langle {\mathbf {L}}_z\rangle /\langle U\rangle$ for the two structures analyzed, SGFZP and LGFZP, over a wide range of focal lengths, the points being the values obtained with FDTD, which shows a good correlation with the model proposed. In the case of SGFZP, a high SAM to OAM conversion is observed for the lower focal lengths, with a maximum conversion factor of about 50% for $\hbox {F}=3\lambda$. In contrast, when the focal length increases the value of $\langle {\mathbf {S}}_z\rangle /\langle U\rangle$ approaches 1 and the value of $\langle {\mathbf {L}}_z\rangle /\langle U\rangle$ tends towards zero; so a very low SAM to OAM conversion is observed for SGFZP working in the far field. This result coincides with the characteristics of the SAM to OAM conversion observed in focusing systems^8–11^, which reaches values near 50% only for high numerical aperture systems. For the designed LGFZP, the $\langle {\mathbf {S}}_z\rangle /\langle U\rangle$ becomes negative for all the focal lengths, starting from $-0.5$ for the lower values, and decreasing asymptotically to $-1$ when the focal length of the structure increases. Therefore, the OAM integral ratio has a value of about 1.5 for $\hbox {F}=3\lambda$, which corresponds to a conversion factor of 150%, and increases asymptotically to 2 as the focal length increases; so conversion factors near 200% can be obtained for far field focusing structures. If we employ the convention used in Q-plates to measure the efficiency, as the ratio between the reached value for OAM and the maximum allowed value (2 in this case), we obtain efficiencies over 98% for focal lengths greater than 300$\lambda$. In the case of high NA, the conversion efficiencies of LGFZP are about 80%, which can be explained by the competition between both mechanisms than explain the SAM to OAM conversion detailed in the introduction. Our system is designed to generate an anisotropy with azimuthal symmetry that causes the conversion, but for high NA aperture structures, the non-paraxial behaviour of the fields also play a role. For this second mechanism the conversion is mainly due to the z component of the electric field (non-paraxial behavior), which only contributed to the orbital angular momentum with a topological charge of 1 (see Eq. 31)and limits the conversion that can be reached. As the NA decreases the z component of the electric field vanishes and the conversion efficiency increases. Therefore, the designed GFZP structures generate an extraordinary SAM to OAM conversion, much greater than the one described for standard high numerical aperture focusing systems, and with the special characteristic that the conversion factor is greater for higher focal distances, while the conversion for the other focusing systems vanishes.

## Conclusions

We have studied the ability of modified binary Fresnel zone plates to change the electromagnetic properties of the incident fields, including an extraordinary spin to orbital angular momentum conversion. This change in the electromagnetic properties was analyzed with FDTD methods and an analytical model was developed using a combination of guided mode theory for the transmittance of the GFZP as a function of the width and depth of the rings, and Stratton–Chu diffraction theory for the free space propagation after the structure. The results show that with the adequate selection of the dimensions great changes in the electromagnetic properties can be obtained. Thus a total inversion of the helicity of fields can be observed, along with SAM to OAM conversion ratios over 150% for high numerical aperture, greater than the conversion factor of 50% observed for other focusing systems. Moreover, the conversion factor for the designed structure reaches values near 200% for higher values of focal distances, which implies conversion efficiencies over 98%, while the standard focusing systems shows very low conversion levels.
